# Supplementary material for: Content-rich biological network constructed by mining PubMed abstracts
Source: BMC Bioinformatics. 2004 Oct 8;5:147. doi: 10.1186/1471-2105-5-147 (PMC528731; doi:10.1186/1471-2105-5-147)
Supplement: Additional File 5 — The original Chilibot query results of the term "long-term potentiation (LTP)" and 22 other terms, limiting the latest references analyzed to the years 1990, 1995, 2000, and 2004. [file 1471-2105-5-147-S5.bz2 › chilibotAdditionalFile5/ltp2004/html/PLC_PKC.html]

 


 **PLC** and **PKC** 
  
Found 710 abstracts in PubMed,  **30 abstracts were retrieved and analyzed**.  


---

 Search Google  |
 PDF files only 
|  EDU domain only 

---

**Interactive relationship** (e.g. stimulation, inhibition, etc)

- Taken together, our results suggest that a SHPS 1  **PLC**  gamma complex activate the  **PLC**   **PKC**  cascade, which is required for the activation of IL 1beta dependent Erk and Akt signalings and cell proliferation.  Ref: 14612947 Int J Oncol, 2003
- In oocytes expressing a pleckstrin homology domain of  **PLC**  delta tagged with GFP, hypo osmotic challenge or  **PKC**  activation caused a translocation of the fluorescence signal from the cell membrane to the cytosol, reflecting PIP 2 hydrolysis.  Ref: 14660621 J Biol ChemJ Biol Chem, 2003
- 2 gliclazide treatment produces a gradual translocation of the diacylglycerol DAG dependent isoforms protein kinase C  **PKC**  alpha, theta, and epsilon from cytosolic to membrane fraction that is dependent on PI3 kinase and phospholipase C  **PLC**  gamma activation.  Ref: 14563700 Endocrinology, 2004
- The transduction pathway of the CD40 receptor may involve the activation of phospholipase C  **PLC**  which induces the production of inositol trisphosphate IP 3 leading to the increase of the intracellular free calcium on one hand, and of diacylglycerol DAG which stimulates the translocation to the membrane of protein kinase C  **PKC** .  Ref: 14568189 Clin Chim Acta, 2003

- :-)

  **Parallel relationship** (e.g. studied together, co-existance, homology, etc.)

  - 24R, 25 OH 2 D 3 dependent  **PKC**  was mediated by phospholipase D... as noted previously for rat RC cells treated with 24R, 25 OH 2 D 3.  Ref: 14635194 J Cell Biochem, 2003
  - NECA mediated IL 6 release was inhibited by the  **PLC**  inhibitor 1 6 17beta 3 methoxyestra, 5 10 tiene 17 yl amino hexyl 1H pyrrole dione, the PI3 kinase inhibitor wortmannin and the  **PKC**  inhibitors bisindolylmaleimide 1 and bisindolymaleimide X1 HCl Ro 32 0432.  Ref: 14504137 Br J Pharmacol, 2003
  - Treatment wit h TNF alpha induced the activities of PC  **PLC**  and  **PKC**  and enhanced NO production.  Ref: 14722058 J Biol ChemJ Biol Chem, 2003
  - These effects were due to inhibition of 3 H DAG production and  **PKC**  activation, implying interference with  **PLC** .  Ref: 14644750 Am J Physiol Renal PhysiolAm J Physiol Renal Physiol, 2003
  - LPA and S1P induced ERK phosphorylation was dependent on the activation of mitogen activated protein kinase MAPK, phospholipase C  **PLC** , and protein kinase C  **PKC** , but was insensitive to pertussis toxin PTX.  Ref: 14648542 Glia, 2004
  - These results demonstrate that BDNF functions at the spinal level to enhance synaptic efficacy in an NMDA receptor dependent manner and requires the action of the  **PLC**   **PKC**  pathway.  Ref: 14622147 Eur J Neurosci, 2003
  - ET 1 through an atypical ET A or ET B receptor activated the  **PLC**   **PKC**  signalling pathway as well as the cAMP pathway.  Ref: 14759557 Regul PeptRegul Pept, 2003
  - However, redistribution of  **PLC**  gamma2 protein occurred in the presence of TPA, no matter whether  **PKC**  inhibitor existed or not.  Ref: 14606067 World J Gastroenterol, 2003
  - and 3  **PKC**  and  **PLC**  gamma activation is necessary for gliclazide stimulated glucose transport.  Ref: 14563700 Endocrinology, 2004
  - Pharmacological inhibition of the  **PLC**   **PKC**  cascade by using specific inhibitor for  **PLC**  gamma U73122 and  **PKC**  GFX strongly inhibited IL 1beta induced Erk and Akt activation.  Ref: 14612947 Int J Oncol, 2003
  - These results taken together suggest that lysophospholipid receptor activation involves receptor coupling to heterotrimeric Gq subunits with consequent activation of  **PLC** ,  **PKC** , and MAPK pathways leading to ERK phosphorylation.  Ref: 14648542 Glia, 2004
  - Cl IB MECA mediated increases in cAMP accumulation suggest that A 3 AR induced ERK1 2 phosphorylation involves adenylyl cyclase activation via phospholipase C  **PLC**  and  **PKC**  stimulation.  Ref: 14751870 Br J Pharmacol, 2004
  - To assess the  **PLC**  activity underlying carbachol induced calcium i oscillations in single HEK293 cells, we co imaged calcium i with fluorescent fusion proteins of protein kinase C  **PKC**  isotypes and the PH domain of  **PLC**  delta1  **PLC**  delta1 PH.  Ref: 14670369 Cell Calcium, 2004
  - Using blockers of  **PKC**  and of phospholipase C  **PLC**  and a mutated AngII receptor lacking Gq coupling, we demonstrate that the signaling pathway of the enhancing effect includes the activation of Gq,  **PLC** , and  **PKC** .  Ref: 14722109 J Biol ChemJ Biol Chem, 2004
  - On the other hand, when cells were treated with  **PKC**  inhibitor alone,  **PLC**  gamma2 protein was still located in the cytoplasm.  Ref: 14606067 World J Gastroenterol, 2003
  - However, TCR induced ERK activation was suppressed by inhibitors of  **PKC**  and  **PLC**  gamma.  Ref: 14680820 Biochem Biophys Res Commun, 2003
  - These findings indicate that adenosine can stimulate IL 6 secretion in FS cells via the A2b receptor coupled principally to  **PLC**   **PKC**  and p38 MAPK.  Ref: 14504137 Br J Pharmacol, 2003
  - PI3 kinase products could induce  **PLC**  gamma activation, whose hydrolytic activity could activate the DAG dependent isoforms  **PKC**  alpha, theta, and epsilon.  Ref: 14563700 Endocrinology, 2004
  - Moreover, TNF alpha induced mPGES 1 by stimulating PC  **PLC**   **PKC**  NO cGMP PKG signal transduction pathway.  Ref: 14722058 J Biol ChemJ Biol Chem, 2004
  - Assembly of C5b 9 increases cytosolic calcium concentration, and results in transactivation of receptor tyrosine kinases, and activation of phospholipase C  **PLC**  gamma1 and the diacylglycerol DAG protein kinase C  **PKC**  pathway.  Ref: 14644750 Am J Physiol Renal PhysiolAm J Physiol Renal Physiol, 2004
  - Thus, Src cascades appear to be coupled to the low affinity CCK A receptor and utilize G q  **PLC**  pathways for their activation, independent of  **PKC**  and CaMK cascades.  Ref: 14741724 Biochem Biophys Res Commun, 2004
  - U 73122 a  **PLC**  inhibitor, wortmannin a PI3 K inhibitor, PD 98059 an ERK inhibitor, Gö 6976 a  **PKC**  inhibitor and Bay 11 7082 a NF kappaB inhibitor significantly decreased LTA stimulated oxidative burst in heterophils by 77%, 30%, 36%, 78%, and 61%, respectively.  Ref: 14555292 Int Immunopharmacol, 2003
  - A pharmacological approach was used to implicate PC  **PLC**   **PKC**  NO signaling as being important for the induction of mPGES 1 by TNF alpha.  Ref: 14722058 J Biol ChemJ Biol Chem, 2003
  - Molecular dissection of the underlying signaling mechanism by a combined biochemical, pharmacological, and genetic approach demonstrates that this BCR controlled integrin mediated adhesion requires the consecutive activation of Lyn, Syk, phosphatidylinositol 3 kinase, Bruton s tyrosine kinase Btk, phospholipase C  **PLC**  gamma2, IP3R mediated calcium release, and  **PKC** .  Ref: 14610042 J Exp MedJ Exp Med, 2003
  - BK stimulated responses were attenuated by inhibitors of selective B 2 receptor Hoe 140, phosphatidylinositol PI  **PLC**  U73122, an intracellular calcium chelator BAPTA AM,  **PKC**  GF109203X, tyrosine kinase genistein, and MEK1 2 PD98059.  Ref: 14751540 Cell Signal, 2004
  - In the present study, we used selective pharmacological inhibitors to investigate the roles of phosphatidylinositol 3 kinase PI3 K, phospholipase C  **PLC** , calcium dependent protein kinase C  **PKC** , extra cellular signal regulated kinase ERK, and nuclear translocation factor kappa B NF kappaB on TLR2 mediated oxidative burst.  Ref: 14555292 Int Immunopharmacol, 2003
  - Data in the present report suggest the participation of a phosphatidylinositol specific  **PLC**  PI  **PLC**  protein kinase C  **PKC**  pathway as the molecular mechanism of DABK mediated stimulation of the Na ATPase activity since i 10 8 M DABK activates PI  **PLC**  activity.  Ref: 14556961 Regul Pept, 2003
  - The  **PLC**   **PKC**  cascade is required for IL 1beta dependent Erk and Akt activation their role in proliferation.  Ref: 14612947 Int J Oncol, 2003
  - Additionally, inhibiting phospholipase C  **PLC**  or protein kinase C  **PKC**  prior to BDNF application completely blocked facilitation.  Ref: 14622147 Eur J Neurosci, 2003
  - These results demonstrate that Ang II produces an inhibition of I Kv in CATH.a cells, via an intracellular pathway that includes  **PLC** ,  **PKC** , and CaMKII.  Ref: 14550259 Biochem Biophys Res Commun, 2003
  - Activated TLR2 utilizes PI3 K,  **PLC** ,  **PKC** , ERK, and NF kappaB as signaling factors that mediate the oxidative burst of chicken heterophils.  Ref: 14555292 Int Immunopharmacol, 2003
  - As noted previously for rat growth plate chondrocytes, 1alpha, 25 OH 2 D 3 mediated its increases in  **PKC**  and  **PLC**  activities in the VDR GC cells through activation of phospholipase A 2 PLA 2.  Ref: 14635194 J Cell Biochem, 2003
  - Receptor and kinase activation is linked to distal signaling pathways  **PLC**  gamma1 activation, calcium influx,  **PKC**  activation and Ras Erk activation via the adapter protein LAT Linker for Activation of T cells.  Ref: 14696041 Bioessays, 2004
  - Tyrosine kinase inhibitor genistein, phosphatidylcholine specific phospholipase C PC  **PLC**  inhibitor D 609 and  **PKC**  inhibitor GF109203X attenuated TNF alpha induced COX 2 expression and PGE 2 synthesis in HTSMCs.  Ref: 14751545 Cell Signal, 2004
